# Bone morphogenetic proteins and the polycystic ovary syndrome

**DOI:** 10.1186/1757-2215-6-32

**Published:** 2013-04-30

**Authors:** E Leonie AF van Houten, Joop SE Laven, Yvonne V Louwers, Anke McLuskey, Axel PN Themmen, Jenny A Visser

**Affiliations:** 1 Department of Internal Medicine, Room Ee532, Erasmus MC, P.O Box 2040, Rotterdam, CA 3000, The Netherlands; 2 Division of Reproductive Medicine, Department of Gynecology and Obstetrics, Erasmus MC, Rotterdam, The Netherlands

**Keywords:** BMPs, PCOS, Marker, Assay

## Abstract

**Background:**

Polycystic Ovary Syndrome (PCOS) is defined by two out of the following three criteria being met: oligo- or anovulation, hyperandrogenism, and polycystic ovaries. Affected women are often obese and insulin resistant. Although the etiology is still unknown, members of the Transforming Growth Factor β (TGFβ) family, including Bone Morphogenetic Proteins (BMPs) and anti-Müllerian hormone (AMH), have been implicated to play a role. In this pilot study we aimed to measure serum BMP levels in PCOS patients.

**Methods:**

Twenty patients, fulfilling the definition of PCOS according to the Rotterdam Criteria, were randomly selected. Serum BMP2, -4, -6 and −7 levels were measured using commercially available BMP2, BMP4, BMP6 and BMP7 immunoassays.

**Results:**

Serum BMP2, serum BMP4 and serum BMP6 levels were undetectable. Three patients had detectable serum BMP7 levels, albeit at the lower limit of the standard curve.

**Conclusions:**

BMP levels were undetectable in almost all patients. This suggests that with the current sensitivity of the BMP assays, measurement of serum BMP levels is not suitable as a diagnostic tool for PCOS.

## Introduction

Polycystic Ovary Syndrome (PCOS) is one of the most common endocrine disorders in premenopausal women [1]. The diagnosis is based on two of the following three criteria being met: hyperandrogenism, oligo- or anovulation, and polycystic ovaries [2]. PCOS is the most frequent cause of infertility in women in their reproductive years, affecting 6-8% of women in the general population worldwide. Besides the reproductive phenotype, women with PCOS often display a metabolic phenotype: 38-88% of women with PCOS are obese with a characteristic abdominal distribution of fat and up to 70% are insulin resistant and some are found to have type II Diabetes Mellitus [3,4]. Although the etiology is unknown, it is generally agreed that elevated androgens are the main culprit of the syndrome [5]. The increased ovarian androgen production may result from the increased GnRH pulsatility, which leads to increased LH secretion in favor of FSH [6]. Combined with the advanced LH responsiveness of small growing follicles, this causes increased androgen production, which is further enhanced by the elevated insulin levels. This abnormal endocrine environment has been suggested to suppress FSH action and causing follicular arrest [7]. In addition, intrinsic alterations in folliculogenesis have been proposed to contribute to the failure in dominant follicle selection in PCOS [7]. Ovarian growth factors, such as members of the transforming growth factor β (TGFβ) family, play important roles in follicle recruitment, follicle selection, and FSH responsiveness. Studies performed predominantly in rodents showed that the various Bone Morphogenetic Proteins (BMPs) are expressed in a cell-specific manner in the ovary, and display spatial and temporal changes in expression depending on the stage of follicular development [8-10]. BMP15 is specifically expressed by oocytes [11], also in ovine, bovine and human, whereas BMP6 has an oocyte/granulosa cell expression pattern in various species. BMP2 is expressed by granulosa cells in rodents and bovine, while BMP4 and BMP7 are theca cells derived growth factors with mRNA expression detectable from the small preantral stage onwards in rats [8,12]. However, in mouse, human and bovine ovaries also granulosa cell expression of BMP4 mRNA has been reported [13-15]. Anti-Müllerian hormone (AMH) is specifically expressed by the granulosa cells of small growing follicles in various species including human [16]. BMPs and AMH differently regulate FSH responsiveness and FSH-induced steroidogenesis, hence it has been suggested that these factors may contribute to the pathogenesis of PCOS.

Several studies have shown that serum AMH levels are elevated in women with PCOS. These elevated levels reflect the increased antral follicle count (AFC) in PCOS. In addition, AMH production per granulosa cell appears to be increased [17-19]. Whether serum BMP levels are altered in women with PCOS is unknown.

Given the role of BMPs in FSH-responsiveness and FSH-induced steroidogenesis, we investigated whether serum BMP levels could be used as a diagnostic tool for PCOS.

## Materials and methods

### Patients

Twenty normogonadotrophic normoestrogenic dysovulatory patients, fulfilling the definition of PCOS according to the Rotterdam Criteria [2], were selected from the Rotterdam PCOS cohort, which comprises of PCOS patients attending our fertility clinic between 1997 and 2011. Standardized initial screening (clinical investigation, transvaginal ultrasound, and fasting blood withdrawal) was performed on a random cycle day between 0900 and 1100 h, irrespective of the interval between blood sampling and the preceding bleeding, as previously described [20]. Biochemically hyperandrogenemia was defined as an elevated (>4.5) free androgen index (testosterone × 100/ SHBG) and clinical hyperandrogenemia was defined as a Ferriman Gallway score > 8. Polycystic ovaries were defined as 12 or more follicles (measuring 2–9 mm) per ovary, and/or an ovarian volume above 10 ml [21]. Endocrine screening included assessment of serum AMH, testosterone and SHBG levels and were determined previously [22,23]. Briefly, blood samples were stored at −20 C until further assessments were made. Serum hormone levels were assessed at the time patients were originally seen. Serum SHBG was measured by luminescence-based immunometric assays (Immulite 2000, Diagnostic Products Corp., Los Angeles, CA). Serum testosterone was measured using a RIA (Diagnostic Products Corp.). AMH levels were measured collectively in samples stored, using an in-house AMH ELISA assay (commercially available through Beckman Coulter, Woerden, The Netherlands). Approval by the local medical ethics committee was obtained and all participants have given informed consent.

### BMP measurements

Serum BMP2, -4 and −7 levels were measured using a Quantikine BMP2, BMP4 and BMP7 Immunoassay (R&D Systems, Minneapolis, MN, USA). Serum BMP6 levels were measured using a human BMP6 DuoSet ELISA Development kit (also R&D Systems). All samples were measured in single measurements according to the manufacturer’s instructions. For BMP2 the detection limit of the assay was between 62.5 pg/mL and 4000 pg/mL. For BMP4 and −7 the detection limits of the assay were between 31.2 pg/mL and 2000 pg/mL. For BMP6 the detection limit of the assay was between 78.13 pg/mL and 1000 pg/mL. Controls with low, medium and high concentrations for BMP2 (207–558 pg/mL, 759–1509 pg/mL, 1670–3061 pg/mL), BMP4 (89–140 pg/mL, 512–780 pg/mL, 958–1468 pg/mL) and BMP7 (180–248 pg/mL, 502–698 pg/mL, 1010–1442 pg/mL) were provided by R&D Systems. For BMP6 controls were not available. All samples where measured in one plate per assay.

## Results

PCOS patient characteristics are given in Table 1. Serum BMP7 was detectable in only three of the 20 PCOS patients (Table 1), albeit at very low levels. Serum BMP2, -4 and −6 were undetectable in all PCOS patients (Table 1). Extending the standard curve with an extra dilution step, allowed the detection of serum BMP4 in only one patient, whereas BMP2 and −6 remained undetectable. In contrast, serum AMH levels were easily detectable at an average concentration of 20.9 ng/mL. For four patients AMH levels were unknown, because data and serum of these patients were collected before AMH measurements were routinely determined in the clinic.

**Table 1:** Clinical characteristics and BMP levels in PCOS women

**Patient**	**Age (years)**	**FAI**	**BMI (kg/m**^**2**^**)**	**AFC**	**AMH (ng/ml)**	**BMP2 (pg/mL)**	**BMP4 (pg/mL)**	**BMP6 (pg/mL)**	**BMP7 (pg/mL)**
*1*	28	1.63	21.6	42	13.2	-	-	-	-
*2*	28	0.92	22	55	28.7	-	-	-	-
*3*	30	1.68	20	47	15.8	-	-	-	-
*4*	20	0.62	22.2	50	20	-	-	-	-
*5*	30	1.03	19.6	59	31.9	-	-	-	57.60
*6*	30	7.93	24.6	40	30.9	-	-	-	-
*7*	23	5.53	19.4	73	28.5	-	-	-	-
*8*	22	5.70	21.1	54	n.d.	-	-	-	-
*9*	20	5.50	24.7	62	n.d.	-	-	-	43.88
*10*	25	13.27	24.2	48	26.6	-	-	-	-
*11*	25	1.50	30.4	32	6.5	-	-	-	-
*12*	31	1.75	44.1	77	23.5	-	-	-	-
*13*	31	1.42	30.1	41	8.8	-	-	-	-
*14*	24	1.21	31.8	44	13.6	-	-	-	-
*15*	26	13.50	30.1	160	n.d.	-	-	-	-
*16*	25	15.13	32.3	153	37	-	-	-	42.38
*17*	27	19.20	32.7	110	n.d.	-	-	-	-
*18*	27	9.20	32.0	103	17.8	-	-	-	-
*19*	28	9.50	34.3	101	n.d.	-	-	-	-
*20*	23	26.57	43.2	91	26.6	-	-	-	-

FAI: Free Androgen Index; BMI: Body Mass Index; AFC: Antral Follicle count.

n.d.: not determined.

- : below detection limit of the assay.

## Discussion

In this pilot study, BMP2, -4 and −6 were undetectable in all PCOS patients. BMP7 was detectable in only three patients, but with levels close to the lower limit of the standard curve. The addition of an extra lowest point to the standard curve did not improve the measurement of BMPs. The patients with discernible serum BMP7 values did not show a consistent pattern with respect to FAI, BMI or AFC. Since the BMPs studied were undetectable in nearly all of the twenty PCOS patients, and therefore could not be used to further distinguish the heterogeneous PCOS population, we did not attempt to analyze a larger number of PCOS patients nor a cohort of normo-ovulatory women as controls.

In a recent study by Son et al. [24], serum BMP4 levels were measured in male and female subjects and shown to be associated with adiposity, insulin resistance and the metabolic syndrome. BMP4 levels ranged between 0.63 ± 0.41 pg/mL and 9.91 ± 4.48 pg/mL and were determined with the same assay as in our study. Since the lowest point of the standard curve of the BMP4 assay is 31.2 pg/mL, these values are well below the standard curve, and therefore it is unclear whether the results of Son et al. have any practical implications. Associations of BMP4 values that far below the detection range are weak at best.

Conflicting results have also been reported for BMP2 and BMP7. Using the same assay, one study showed that serum BMP2 levels were undetectable in patients with femoral fractures [25], whereas in another study, serum BMP2 and −7 levels [10,26,27] could be detected in patients with ankylosing spondylitis, arthritis and healthy subjects [28]. Also, in the latter study BMP levels were near or below the detection range of the assay. In agreement, other studies have also shown that serum BMP7 levels are often below or close to the standard curve of the assay [29,30].

All women used in this study had polycystic ovaries and in accordance with these results increased AMH levels. An explanation for the undetectable serum BMP levels in this study could be that BMPs are not secreted by the human ovary, although it has been reported that BMP2, -4, -6 and 7 are expressed by the human ovary [9]. This explanation may not be likely since this would be in contrast to other ovarian expressed TGFβ family members that are secreted, such as AMH, Inhibin B and Activin A [18,31]. Alternatively, BMP immunoreactivity may not have been preserved. Prolonged storage and repeated freeze/thawing of the samples did not affect the immunoreactivity of AMH, a family member of BMPs, but an effect on BMP immunoreactivity cannot be ruled out. However, based on the studies mentioned above and our own study, we prefer to suggest an alternative reason, namely that the current available BMP assays are not sensitive enough to detect BMP ligands in the circulation of human subjects. Therefore, more sensitive assays are necessary to determine whether serum BMP levels could be used as an additional diagnostic tool in PCOS and other metabolic diseases.

## Competing interests

The authors declare that they have no competing interests.

## Authors’ contributions

ELAFvH performed the experiments, analyzed and interpreted data, and drafted the manuscript. JSEL and YVL acquired patient data and samples, and contributed to the writing of the manuscript. AM performed the experiments. APNT conceived and designed the study, and contributed to the writing of the manuscript. JAV conceived and designed the study, supervised the work and co-wrote the manuscript. All authors have read and approved the final manuscript.
